# Effectiveness of Combining Organizational Alcohol Policy and Skills Training for Managers to Reduce Hazardous Alcohol Consumption in Swedish Workplaces: Study Protocol for a Cluster Randomized Study

**DOI:** 10.2196/17145

**Published:** 2020-08-12

**Authors:** Devy Lysandra Elling, Martina Wilson, Per Carlbring, Peter Wennberg, Kristina Sundqvist

**Affiliations:** 1 Department of Public Health Sciences Stockholm University Stockholm Sweden; 2 Department of Psychology University of Gothenburg Gothenburg Sweden; 3 Department of Psychology Stockholm University Stockholm Sweden; 4 Department of Global Public Health Karolinska Institutet Stockholm Sweden

**Keywords:** alcohol prevention, health promotion, workplace intervention, hazardous alcohol use, alcohol use, intervention, workplace

## Abstract

**Background:**

High alcohol consumption poses risks to individual health and society. Previous alcohol interventions have mainly focused on high-risk consumers or young adults in school-based settings. Since the majority of the adult population is in the workforce, the workplace can be considered a favorable arena for implementing interventions.

**Objective:**

This protocol describes a project aimed at increasing knowledge of the effectiveness of combining the implementation of an organizational alcohol policy with skills training for managers as a workplace alcohol prevention program, by evaluating the intervention and exploring managers’ perceptions of the intervention.

**Methods:**

Organizations with at least 100 employees were invited to take part in the project. A total of 11 organizations (744 managers and 11,761 employees) were included in the project. Data are collected through self-administered online surveys at baseline, 12 months, and 24 months. The primary outcome is managers’ inclination to initiate an early alcohol intervention (eg, by initiating a dialogue) when concern regarding employees’ hazardous alcohol consumption arises. The secondary outcomes of interest are managers’ and employees’ organizational alcohol policy knowledge and changes in alcohol consumption, as measured using the Alcohol Use Disorder Identification Test (AUDIT) score. A linear mixed-model framework will be used to model variability on different levels. Primary analysis will follow an intention-to-treat approach. Additionally, managers’ responses from semistructured interviews will be analyzed using thematic analysis to explore managers’ experiences regarding the prevention program.

**Results:**

This study is ongoing. The overall study start was on January 2018, and the study is planned to end in December 2020. Baseline and 12-month follow-up measurements have been collected.

**Conclusions:**

This project is designed to evaluate the effectiveness of an alcohol prevention program regarding higher inclination to initiate early alcohol interventions after policy implementation and skills training among managers, compared to the usual practices in the workplace. The results from this study can contribute to increased knowledge about alcohol interventions and future prevention programs in the workplace.

**Trial Registration:**

ISRCTN17250048; http://www.isrctn.com/ISRCTN17250048

**International Registered Report Identifier (IRRID):**

DERR1-10.2196/17145

## Introduction

High alcohol consumption poses risks not only to individual health [1-3] but it may also lead to detrimental effects in the society [4,5]. In Sweden, although a decrease in alcohol consumption is observed among the younger population, older adults have shown the opposite trend over the past few decades [6,7]. For example, the Public Health Agency of Sweden reported that 13% of women and 20% of men (>16 years old) are classified as hazardous alcohol consumers [6].

The majority of existing interventions have been conducted in health care settings and social services, which often focus on high-risk individuals aiming at reducing their alcohol consumption [8,9]. However, since the majority of adults neither consume a harmful amount nor have an alcohol dependence, those with a low to moderate consumption are often overlooked [10]. Some of the adverse effects include increased rate of injuries [1] and economic losses (absenteeism and presenteeism) [11,12]. Given the costs stemming from high alcohol consumption, the workplace may be an appropriate setting for preventive strategies. However, previous studies often focused on young adults in school-based settings [13,14], and workplace-based prevention programs have solely been conducted outside of Sweden [5,15-17].

Considering that workers with high alcohol consumption are often identified at a later stage, such as after the occurrence of adverse effects, many workplace-based interventions employ brief intervention (BI) strategies aiming to reduce alcohol consumption. Some workplaces conduct BI with various methods, including motivational approaches and personalized feedback [18-20], which are often combined with alcohol screening. Moreover, some workplaces conduct alcohol screening or monitoring prior to or during employment. Although screening and monitoring can reduce harmful alcohol consumption, it only shows effectiveness in the short-term. Conversely, an organizational alcohol policy could be effective in the long-term, especially in workplaces with limited resources [16]. However, it may be counterproductive if not implemented properly.

From a public health perspective, a preventive strategy is a focal point of interest for many reasons. Based on Rose’s Prevention Paradox theory — that the effects of a prevention program are greatest when it is targeted at the population at large rather than high-risk individuals [21] — the workplace can help reach hazardous consumers while reducing the risk of stigmatization. Many workplace alcohol preventive interventions aimed at reducing alcohol consumption target high-risk consumers by combining BI and screening, a combination that could be effective in some sectors [18,22]. Based on previous literature, some workplace sectors, such as construction, hospitality, and transport sectors, have an overrepresentation of high alcohol consumption [23]. Therefore, it is of interest to examine whether these sectors would benefit from an alcohol preventive intervention targeting the whole workplace. For instance, a recent study conducted in Australian manufacturing organizations investigated whether a multicomponent preventive intervention (organizational policy and skills development among managers) could effectively identify hazardous alcohol consumers at an early stage [17]. Even though Pidd et al [17] concluded that the program was not effective in reducing alcohol consumption, it raised awareness of the negative consequences of alcohol consumption, not only for individual’s health but also for the workplace as a whole.

Given that the implementation of an organizational alcohol policy is recommended in Swedish workplaces [24], the combination in terms of improving managers’ skills to identify hazardous alcohol consumption is still deficient. By collaborating with an organization that provides substance-related prevention services to workplaces (Alna), this project evaluates a multicomponent alcohol prevention program, which includes development of an organizational alcohol policy and skills training for managers. Specifically, the prevention program aims to contribute to knowledge about the potential effects of the combination of an organizational alcohol policy and managers’ skills development in the workplace. In addition, we hypothesize that the prevention program will result in (1) managers reporting an increased inclination to initiate an early alcohol intervention when concern or suspicion of hazardous alcohol consumption arises; (2) an increase in employees’ knowledge on organizational alcohol policy, guidelines, and support regarding hazardous alcohol consumption; (3) an increased number of early interventions (eg, initiate a dialogue) to help employees with hazardous consumption of alcohol and other substances; (4) more sustainable alcohol consumption both among managers and employees, as measured using the Alcohol Use Disorder Identification Test (AUDIT) score; (5) a reduction in the number of cases of hazardous alcohol consumption among managers and employees, as measured with the AUDIT score; and (6) increased confidence among managers in handling hazardous alcohol consumption in the workplace.

## Methods

**Figure 1:**
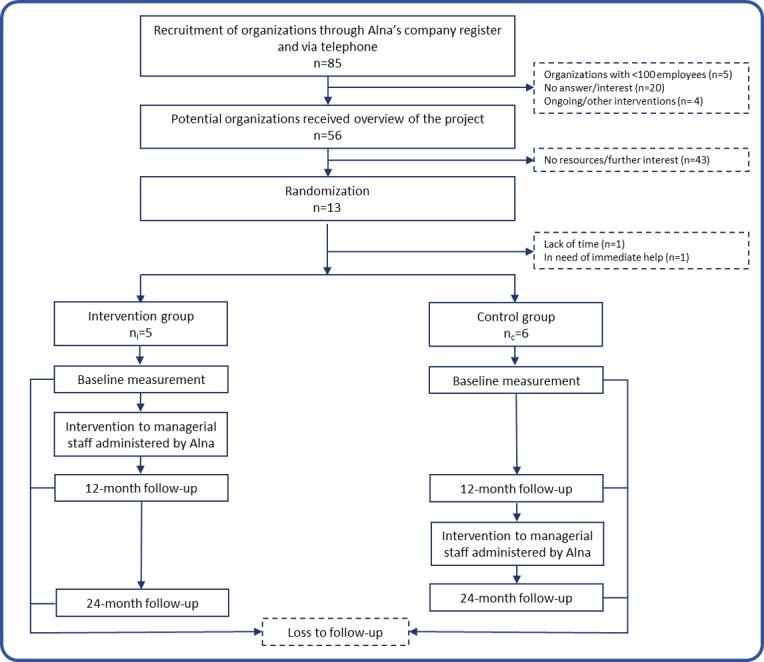
Schematic overview of the study process, from recruitment of participants to follow-up endpoints of the cluster randomized study.

This project is a two-armed cluster randomized study with follow-up at two time points: 12 months and 24 months (Figure 1). The organizations are randomized to an intervention group or a control group. More detailed information on each trial arm can be found in the “Intervention” and “Control Group” sections, respectively.

### Study Population and Recruitment

This project consists of managers (n=744) and employees (n=11,761) from 11 organizations in Sweden. The manager group comprises all individual staff members with delegated staff liability, including supervisors, group leaders, and human resources personnel. All other individuals are classified as employees. Table 1 summarizes the organizational sectors, gender distribution, and age of all staff members in each recruited organization.

The organizations were recruited in two ways: partly by sending information about the study rationale to all organizations with a minimum of 100 employees registered in Alna’s company register (2139 organizations) and partly by contacting representatives from some of the largest organizations in Sweden by telephone and inviting them to participate in the study. In accordance with previous research, the necessity for alcohol prevention programs is greater amongst certain sectors and consequently, the construction, hospitality, and transport sectors were prioritized during the recruitment process of this project [23].

**Table 1:** Overview of each organization’s staff members.

ID	Sector	Number of staff members^a^	Female, n (%)	Age (years), mean
1	Brewery	153	49 (32.2)	41.1
2	Insurance	1628	707 (43.4)	37.6
3	Security	1963	538 (27.4)	38.9
4	Hospitality	521	314 (60.3)	36.8
5	Transportation	781	337 (43.1)	43.0
6	Nursing care	429	344 (80.2)	42.0
7	Security	5000^b^	N/A^c^	N/A^c^
8	Construction	1066	209 (19.6)	41.1
9	Hospitality	685	333 (48.6)	32.8
10	Transportation	164	15 (9.1)	44.0
11	Security	111	15 (13.5)	N/A^c^

^a^The number of staff members reflects the population of managers and employees at the time of recruitment.

^b^Number of individuals was approximated.

^c^N/A: not available because the information was not provided by the organization.

During the recruitment process, 13 organizations expressed their interest. An overview of the study, including information about its relevance, was sent to the recruited organizations. Two of the organizations were dissatisfied with the group allocation and dropped out.

Information about the study procedure was provided to the participants, both at the organizational and individual levels. In terms of individual-level consent, information about the study and a statement of consent provision were presented prior to the start of the survey. The consent statement informed the participants that commencing the survey was considered as consent to participate in the study. See Multimedia Appendix 1 for an English translation of the information presented at the beginning of the survey.

### Randomization

In order to avoid contamination between the intervention and control groups, the organizations were randomized at the organizational level through block randomization. The organizations were matched based on type of sector (eg, hospitality sector) and size of organization in blocks of 2-4 organizations. Each block was allocated to either the intervention or control group by an online web service (random.org).

### Blinding

Due to the nature of the study and assessment of the waitlist condition, it was not possible to blind either the researchers or the managers in each organization. However, the surveys are administered online without any involvement of the researchers. Hence, the survey is not subject to interpretation bias from the researchers.

### Trial Arms

#### Intervention Group

Based on Alna’s previous experience [25] and framework, the prevention program comprises two components: implementation of an organizational alcohol policy and skills development training.

The first component focuses on the implementation of an organizational alcohol policy. Alna is assisting managers in improving and implementing an organizational alcohol policy, where the policy is based on Alna’s previous experiences. This includes examples of responsibility and an operational plan in which strategies, reasons for action, person responsible, timeframe, and required resources are included. The alcohol policy was developed together with human resources personnel and management on 3-4 occasions, each lasting for approximately 2 hours depending on the availability of the organization. In addition, the alcohol policy was tailored to each organization and aligned with their organizational values.

The second component of the program is skills development training, with the purpose of helping managers identify early signs of hazardous alcohol consumption and act upon behaviors that may lead to adverse effects for both employees and the organization. Managers attend 2 training workshops directed by Alna, which last for 3.5 hours per session. The workshops cover various topics regarding addiction, prevention, and dialogue about the hazardous use of alcohol. At the end of the second session, a “checklist for managers regarding alcohol use” is introduced (Textbox 1).

**Topics covered during the skills development workshops**

1. Types of alcohol use: differences between risk consumption, harmful consumption, and alcohol dependence
2. Prevalence: statistics on prevalence and trends over time regarding alcohol use and hazardous alcohol consumption in Sweden
3. Risk factors: risk and protective factors of hazardous alcohol consumption in the workplace
4. Initiating a dialogue: the importance of dialogue as a tool; preparation, conducting, and evaluation of a dialogue regarding potential problematic drinking
5. Signals: behaviors and signs indicating hazardous alcohol consumption
6. Workplace culture and policy: the role of workplace culture in contributing to or preventing hazardous alcohol consumption; benefits of implementing workplace policies
7. Roles and responsibilities: discussion of roles and responsibilities of the organization involving managers and employees
8. Use of an implementation checklist: participants go through an implementation checklist and its purpose
9. Dilemma: discussion about ambiguous cases

#### Control Group

Organizations in the control group are placed on a waitlist and continue their usual practices. The control group receives the same prevention program as the intervention group after the 12-month follow-up. Individuals in the control group respond to the survey parallel to individuals in the intervention group.

### Data Collection

Data are collected through self-reported online questionnaires. The organizations have provided Alna with a list of emails and demographics of the staff. The surveys are distributed using email, SMS text messaging, or a general link. Participants with email addresses and phone numbers receive a unique link to the survey, which reduces the probability of the data being wrongly coded or disappearing. The participants who do not have or do not provide an email address or telephone number receive a general link to the survey via their workplace’s internal website. In order to follow up with the participants with a general link, they are requested to create a code prior to the survey using their initials and the last four digits of their social security number. For example, a fictional person, Anna Carlsson with social security number 19520824-5982 would enter AC5982. This ensures that they are able to fill in the same code during the follow-ups without having to remember or store anything.

Two surveys were created: one for managers and one for employees. Both surveys include questions regarding demographics, knowledge of organizational alcohol policies, and alcohol habits measured using the AUDIT. The survey for managers also includes questions regarding managerial responsibility and experience and their inclination to initiate early alcohol interventions, such as initiating a dialogue with their employees. The AUDIT segment of the survey is based on the previously validated World Health Organization screening tools to assess alcohol consumption [26]. The surveys were piloted (n=20) upon completion of the online version of the survey, and appropriate modifications (reformulation of questions and design layout) were completed.

To increase the response rate, 3 reminders at 1-week intervals are sent to participants who provide incomplete answers to the survey.

Managers across the organizations were interviewed using semistructured interviews. Managers’ experiences with the implementation processes and effects of the prevention program will be explored. In order to explore managers’ perceptions of an alcohol prevention program, semistructured interviews were conducted with managers (n=61) in the intervention and control groups. The interviews were conducted after the first follow-up to avoid influence of the outcome measures on the follow-up survey. Participants that expressed interest in being interviewed during the time of the “intervention to the managerial staff administered by Alna” were contacted by telephone or email between September and November 2019. The interviews were conducted via telephone for approximately 40 minutes. The participants provided verbal consent, and the interviews were recorded and transcribed verbatim.

The interview questions and analysis aim to explore respondents’ perceptions and experiences of (1) alcohol problem prevention in the workplace, (2) the educational part of the “intervention to the managerial staff administered by Alna,” (3) difficulties as well as enabling factors associated with implementing an alcohol policy in the workplace, and (4) how to handle a situation in which an employee or co-worker appears to have an alcohol problems. The interview questions were constructed primarily by one of the authors, a psychologist with clinical experience working with addiction.

All interview questions were open-ended to invite managers’ own thoughts and experiences; the aim is to cover their general understanding of phenomena as well as their personal experiences. Follow-up questions were asked in order to ameliorate comprehension of the respondents’ experiences and thoughts, with the number of follow-up questions varying based on how detailed the respondents were in their descriptions. The transcripts will be analyzed qualitatively using thematic analysis. All personal details are coded to ensure anonymity.

### Outcome Measures

The primary outcome of this project is managers’ self-reported inclination to initiate early alcohol interventions, such as initiating dialogue with employees when suspicion or concern about hazardous alcohol consumption arises (outcome 1). This is measured using the following items: “To be able to initiate a dialogue about alcohol consumption with an employee, I want to be sure that the person has a problem,” “For an employee to receive help with their alcohol-related issues, the person has to first admit that they have a problem,” and “If an employee has a problem that could be due to alcohol use, I feel confident in initiating a dialogue about it.” A 5-point scale is used, ranging from 1 (strongly disagree) to 5 (strongly agree).

Survey items regarding employees’ knowledge on organizational alcohol policy, guidelines, and available support (outcome 2) are measured using statements rated on a 5-point Likert scale, ranging from 1 (very poorly) to 5 (very well). Managers’ actions to address employees’ alcohol consumption is measured using 2 items with yes/no alternatives (outcome 3). The total AUDIT score is used to assess sustainable alcohol consumption (outcome 4) and number of cases of hazardous alcohol consumption (outcome 5) among managers and employees. To examine confidence among managers, questions regarding managers’ self-perceived knowledge about hazardous alcohol consumption and the way to handle alcohol-related issues are used (outcome 6).

In addition, basic demographic questions, including gender, age, and highest level of education attained are utilized to examine the individual differences within and across organizations.

### Statistical Analyses

The project inherits a hierarchical structure, which violates the assumption of independent samples required for analysis of variance. Therefore, a linear mixed-model framework will be conducted during the statistical analyses to model the variability on different levels (managers and employees) [27]. Each outcome corresponds to the questionnaire question (see Outcome Measures), and changes in the corresponding question from baseline to the follow-ups will be analyzed. Data management and all statistical analyses will be conducted using Stata Statistical Software v.14 (StataCorp, College Station, TX).

### Qualitative Analysis

The interview transcripts will be analyzed using inductive thematic analysis, a method aimed at identifying recurring themes, concepts, or phenomena described by the participants [28]. In inductive analysis, no attempt will be made to fitting data into a pre-existing framework. The qualitative analysis will be conducted using the program Atlas.ti. Transcripts of extracted data are read and coded independently to be able to extract relevant data that may contribute to answering the research questions of the study. After the coding process, each dataset will be reread to ensure that nothing was overlooked. Codes and ideas will be discussed between the authors prior to categorizing extracts and creating themes.

### Attrition

This study will use intention-to-treat analyses to retain information about the participants based on their group allocation and complete case analyses to be able to compare changes in outcome measures for managers and employees who adhere to the prevention program. We expect to be able to perform sensitivity analyses to examine differences between complete samples and respondent samples since organizations are providing some demographic data about their employees. Imputation using maximum likelihood techniques may be applied to avoid distortion of the mean, variance, or covariance to other variables.

### Sample Size

The data will be fitted using a repeated measures hierarchical linear model [29]. We aimed to recruit 10-14 organizations, with approximately 10,000-15,000 participants. Assuming there are no associations between hierarchical levels (ie, managers and employees), the power of this study is calculated based on the total number of participants, yielding a power >95%, given the small effect size (Cohen d <0.20) at 5% significance. In contrast, if all the variance in the study could be explained by the hierarchical level, the power of this study is calculated based on the number of levels, yielding approximately a 69% power given the small effect size with a significance level set at 5%. Presuming that the hierarchical levels explain only some of the variance, the current study design should be sufficient to achieve greater than the conventional norm of 80% power.

## Results

This study is ongoing. Recruitment of organizations was completed between March and May 2017. The overall start was on January 2018 and is planned to end in December 2020. Baseline and 12-month follow-up measurements have been collected. This project was granted an ethical permit by the Ethical Review Board of Stockholm Region (dnr 2018/634-31/5) on April 12, 2018. In 2018, we recruited a total of 12,505 participants (744 managers and 11,761 employees). Basic scientific results of the project have been uploaded on the ISRCTN registry on April 7, 2020 (https://www.isrctn.com/ISRCTN17250048).

## Discussion

This study is designed to evaluate the effectiveness of an alcohol prevention program in the workplace in Sweden. To our knowledge, it is the first study in Sweden targeting individuals in workplace settings. The strategies used to prevent harm to the individuals and organizations will help create evidence-based policies in the workplace. If the program is successful, it will act as a complementary method to a conservative approach to treating high-risk alcohol consumers in the workplace.

This prevention program targets the majority of the adult population instead of high-risk individuals, which is one of the key approaches in the public health field [21]. This study could potentially lead to managers being able to identify and feel more confident in initiating interventions before their employees develop drinking problems that could be hazardous to their health. The results produced in this study could possibly be of high value to other workplace settings that are often not considered to be vulnerable to high alcohol consumption, as suggested by previous literature and organizations included in this project [23].

This study has some limitations. One of its main concerns is the self-reported perception of outcome measures. This may lead to bias, as participants may provide answers that are socially desirable. Considering that policy implementation in this study is specific to each organization, the generalization of such effects to other settings (different types of sectors) and populations (nonworking populations) may be difficult. Further, a low response rate is expected in some of the organizations, given that some participants will receive their questionnaires through a general link. The validity of the study may be negatively affected due to difficulties interpreting the results and comparing them across organizations. In addition, since this project implements and evaluates the prevention program, we are merely able to evaluate the combined effect of the prevention program. Therefore, the effect of one of the intervention components is unknown. Finally, considering that the prevention program is implemented at the organizational level and outcome assessments are conducted on individual level (ie, managerial and employee levels), we are unable to identify organizational factors that may influence the results of this project.

### Ethics and Dissemination

#### Ethical Approval

The Ethical Review Board of Stockholm Region (dnr 2018/634-31/5) granted an ethical permit for this study.

#### Information and Informed Consent

An overview of the project’s rationale was distributed at the organizational level, and a representative from each organization provided organizational level consent. With regard to individual-level consent, an information sheet was distributed by the representatives through the organization’s internal website or newsletter. Moreover, each participant was presented with information about the study and a statement of consent provision at the start of the survey. Therefore, participants were considered to give their consent to participate in the study by starting the survey. Participants’ data are handled according to the General Data Protection Regulation and Stockholm University’s rules and guidelines. In addition, participants are able to decline or withdraw their participation in the study without giving any reason.

#### Dissemination of Results

The results of this study will be presented at conferences and published in peer-reviewed scientific journals.

## Appendix

Multimedia Appendix 1 Consent form.

## Abbreviations

AUDIT: Alcohol Use Disorder Identification Test
BI: brief intervention
