# Supplementary material for: Effectiveness of Combining Organizational Alcohol Policy and Skills Training for Managers to Reduce Hazardous Alcohol Consumption in Swedish Workplaces: Study Protocol for a Cluster Randomized Study
Source: JMIR Res Protoc. 2020 Aug 12;9(8):e17145. doi: 10.2196/17145 (PMC7450387; doi:10.2196/17145)
Supplement: Multimedia Appendix 1 [file resprot_v9i8e17145_app1.pdf]

Hello!

Your workplace is participating in a project about alcohol prevention in the workplace. The project is a collaboration between the Department of Public Health Sciences at Stockholm University and Alna Sweden. The project is financed by the Public Health Agency of Sweden.

We would like to ask you to a few short questions. Participation in the study is voluntary. You do not need to give any motivations if you choose not to, and any information you have provided will be deleted. The questionnaire will take approximately 5-10 minutes. Your answers and results will be kept confidential, and only involved researchers will know your answers. Your employer will not be able to know your specific answers.

Stockholm University is responsible for the research and handling of personal information. The project leader for this study is Dr. Kristina Sundqvist and responsible researcher is Professor Peter Wennberg. If you have any questions or concerns about the project, or how your data is handled, you can send an e-mail to alkoholprevention@su.se.

Your answer is important to us, and we hope that you will participate!

You can read more about the project here:

https://www.su.se/publichealth/forskning/forskningsprojekt/alkoholpreventiva-insatser-i-arbetslivet-en-randomiserad-kontrollerad-studie

Stockholms universitet

ALNA
Expert på skadligt bruk i arbetslivet

Folkhälsomyndigheten
